# A randomized controlled trial to compare a restrictive strategy to usual care for the effectiveness of cholecystectomy in patients with symptomatic gallstones (SECURE trial protocol)

**DOI:** 10.1186/s12893-016-0160-3

**Published:** 2016-07-13

**Authors:** P. R. de Reuver, A. H. van Dijk, S. Z. Wennmacker, M. P. Lamberts, D. Boerma, B. L. den Oudsten, M. G. W. Dijkgraaf, S. C. Donkervoort, J. A. Roukema, G. P. Westert, J. P. H. Drenth, C. J. H. van Laarhoven, M. A. Boermeester

**Affiliations:** Department of Surgery, Radboud University Medical Centre, P.O. Box 9101, 6500 HB Nijmegen, The Netherlands; Department of Surgery, Meibergdreef 9, 1105 AZ Amsterdam, The Netherlands; Department of Gastroenterology, Hepatology, Radboud University Medical Centre, P.O. Box 9101, 6500 HB Nijmegen, The Netherlands; Department of Surgery, St. Antonius Hospital, Koekoekslaan 1, 3435 CM Nieuwegein, The Netherlands; Department of Medical and Clinical Psychology, Tilburg University, P.O. Box 90153, 5000 LE Tilburg, The Netherlands; Clinical Research Unit, Academic Medical Centre, Meibergdreef 9, 1105 AZ Amsterdam, The Netherlands; Department of Surgery, Onze Lieve Vrouwe Gasthuis, Oosterpark 9, 1091 AC Amsterdam, The Netherlands; Department of Surgery, St. Elisabeth Tweesteden Hospital, Hilvarenbeekseweg 60, 5022 GC Tilburg, The Netherlands; Department of IQ Healthcare, Radboud University Medical Centre, P.O. Box 9101, 6500 HB Nijmegen, The Netherlands

**Keywords:** Cholecystectomy, Symptomatic gallstones, Abdominal pain, Cost-effectiveness

## Abstract

**Background:**

Five to 22 % of the adult Western population has gallstones. Among them, 13 to 22 % become symptomatic during their lifetime. Cholecystectomy is the preferred treatment for symptomatic cholecystolithiasis. Remarkably, cholecystectomy provides symptom relief in only 60-70 % of patients. The objective of this trial is to compare the effectiveness of usual (operative) care with a restrictive strategy using a standardized work-up with stepwise selection for cholecystectomy in patients with gallstones and abdominal complaints.

**Design and methods:**

The SECURE-trial is designed as a multicenter, randomized, parallel-arm, non-inferiority trial in patients with abdominal symptoms and ultrasound proven gallstones or sludge. If patients meet the inclusion criteria they will be randomized to either usual care or the restrictive strategy.

Patients in the usual care group will be treated according to the physician’s knowledge and preference. Patients in the restrictive care group will be treated with interval evaluation and stepwise selection for laparoscopic cholecystectomy. In this stepwise selection, patients strictly meeting the preselected criteria for symptomatic cholecystolithiasis will be offered a cholecystectomy. Patients not meeting these criteria will be assessed for other diagnoses and re-evaluated at 3-monthly intervals. Follow-up consists of web-based questionnaires at 3, 6, 9 and 12 months.

The main end point of this trial is defined as the proportion of patients being pain-free at 12 months follow-up. Pain will be assessed with the Izbicki Pain Score and Gallstone Symptom Score.

Secondary endpoints will be the proportion of patients with complications due to gallstones or cholecystectomy, the association between the patients’ symptoms and treatment and work performance, and ultimately, cost-effectiveness.

**Discussion:**

The SECURE trial is the first randomized controlled trial examining the effectiveness of usual care versus restrictive care in patients with symptomatic gallstones. The outcome of this trial will inform clinicians whether a more restrictive strategy can minimize persistent pain in post-operative patients at least as good as usual care does, but at a lower cholecystectomy rate. (The Netherlands National Trial Register NTR4022, 17th December 2012)

**Trial registration:**

The Netherlands National Trial Register NTR4022

http://www.zonmw.nl/nl/projecten/project-detail/scrutinizing-inefficient-use-of-cholecystectomy-a-randomized-trial-concerning-variation-in-practi/samenvatting/

## Background

Five to 22 % of the adult Western population has gallstones [1, 2]. Most patients with gallstones remain asymptomatic. About 13–22 % of the patients with gallstones will eventually become symptomatic [3, 4]. Yearly, this corresponds to approximately 28,000 patients in The Netherlands and over 800,000 patients in the US [5, 6]. The life time risk of complicated cholecystolithiasis causing choledocholithiasis, acute cholecystitis, acute pancreatitis or cholangitis is 5 % [7]. Removal of the gallbladder (i.e. cholecystectomy) is the first choice of treatment in symptomatic cholecystolithiasis [8–10]. Laparoscopic cholecystectomy is associated with 5.5 % morbidity and 0.2 % mortality [11]. Bile duct injury with an incidence of 0.5–1.0 % is the most severe complication [12]. Despite the high number of cholecystectomies performed worldwide, this approach appears to be ineffective for up to 40 % of patients with persistent post-operative pain [13].

The typical presentation of uncomplicated cholecystolithiasis is a biliary colic, consisting of a steady pain, usually located in epigastrium and/or in the right upper quadrant, lasting 30 min or longer [14, 15]. However, the assessment and management of cholelithiasis and cholecystolithiasis varies between surgeons, hospitals and countries mainly due to a lack of evidence-based treatment guidelines [16–18].

Variation in the cholecystectomy rate and differences in indication for surgery are reported [3, 19, 20]. The variety in indications for cholecystectomy is hypothesized to be one of the causes for the substantial practice variation in cholecystectomies in The Netherlands [21].

Dutch health care insurance companies have reported that the variation in cholecystectomies performed per 100,000 insured inhabitants varied from 48 to 262 procedures, a fivefold variation. Because LC is a common surgical procedure mostly performed in regional hospitals, a centralization effect cannot explain this variation. Moreover, the total number of cholecystectomies in the Netherlands increased from 12,000 per year in 1990 to 23,000 per year at present, a 100 % increase, whereas the Dutch population increased from 15 to 17 million people, an increase of 13 % [5]. The indications for cholecystectomy are currently not based on evidence from randomized research and are not restricted within guidelines. The motivation to perform a cholecystectomy is not only dependent on an appropriate clinical indication, but is also influenced by patient preference and the financial incentive to operate. Patients with persistent pain generate an ongoing and significant health economic burden for society. Investigating the right indication for cholecyctectomy is in line with recent initiatives to improve patient health and reduce risks and healthcare overuse. [22, 23]

This trial aims to optimize the outcomes of patients with gallstones and abdominal complaints by identifying which patients benefit most from surgery for symptomatic cholecystolithiasis. This will clarify which patients have an absolute indication for cholecystectomy and minimize the proportion of patients undergoing unnecessary cholecystectomy. In this randomized trial usual care will be compared with a restrictive strategy consisting of a standardized work-up and stepwise selection for cholecystectomy. This study will evaluate if stepwise selection for cholecystectomy is non-inferior to usual care with respect to the proportion of patients being pain-free after 12 months. We hypothesize that stepwise selection of patients with cholecystolithiasis for cholecystectomy is non-inferior to usual care with respect to the patient reported outcome, but attributes to a more appropriate use of care and provides a basis for a lower number of cholecystectomies performed. This may improve patients’ health status, prevent complications, reduce health care demand and, consequently, lower health care costs.

## Methods and design

### Design

The SECURE-trial (Scrutinizing (in)efficient use of cholecystectomy: a randomized trial concerning variation in practice) is designed as a multi-center, randomized, parallel-arm, non-inferiority study in 1038 subjects with abdominal symptoms and ultrasound proven gallstones or sludge. In this multicenter trial 24 centers are included, located throughout The Netherlands.

### Outcomes

The primary endpoint of the SECURE-trial is defined as the proportion of patients being pain- free at 12 months follow-up. Pain and pain medication use will be assessed with the Izbicki Pain Score and Gallstone Symptom Score.

Effectiveness is defined as the proportion of patients being pain-free at 12 months follow-up.

Secondary endpoints will be the proportion of patients with complications due to gallstones or cholecystectomy, the association between the patients’ symptoms and treatment and work performance, and ultimately, cost-effectiveness.

Finally practice variation will also be examined with the variance indicator being defined as the number of patients undergoing cholecystectomy per 100,000 inhabitants in the catchment area of the hospital, adjusted for relevant patient characteristics (i.e. age, sex, socio-economic status).

### Study population

#### Inclusion criteria

The trial will include all patients between the age of 18 and 95 who are referred to a surgical out-patient clinic with ultra-sound proven gallstones or sludge and abdominal complaints.

Exclusion criteria are (a) a history of complicated cholelithiasis (i.e., choledocholithiasis, acute cholecystitis, percutaneous gallbladder drainage, biliary pancreatitis or cholangitis); (b) an indication for primary open cholecystectomy; (c) a history of current malignancy; (d) an expected short life span of less than 12 months; (e) an American Society of Anaesthesiologists physicial status classification (ASA) of III and IV; (f) known liver cirrhosis; (g) cognitive disorders that predispose unreliable questionnaire responses; (h) insufficient knowledge of the Dutch language and (i) pregnancy.

### Study arms

**Fig. 1:**
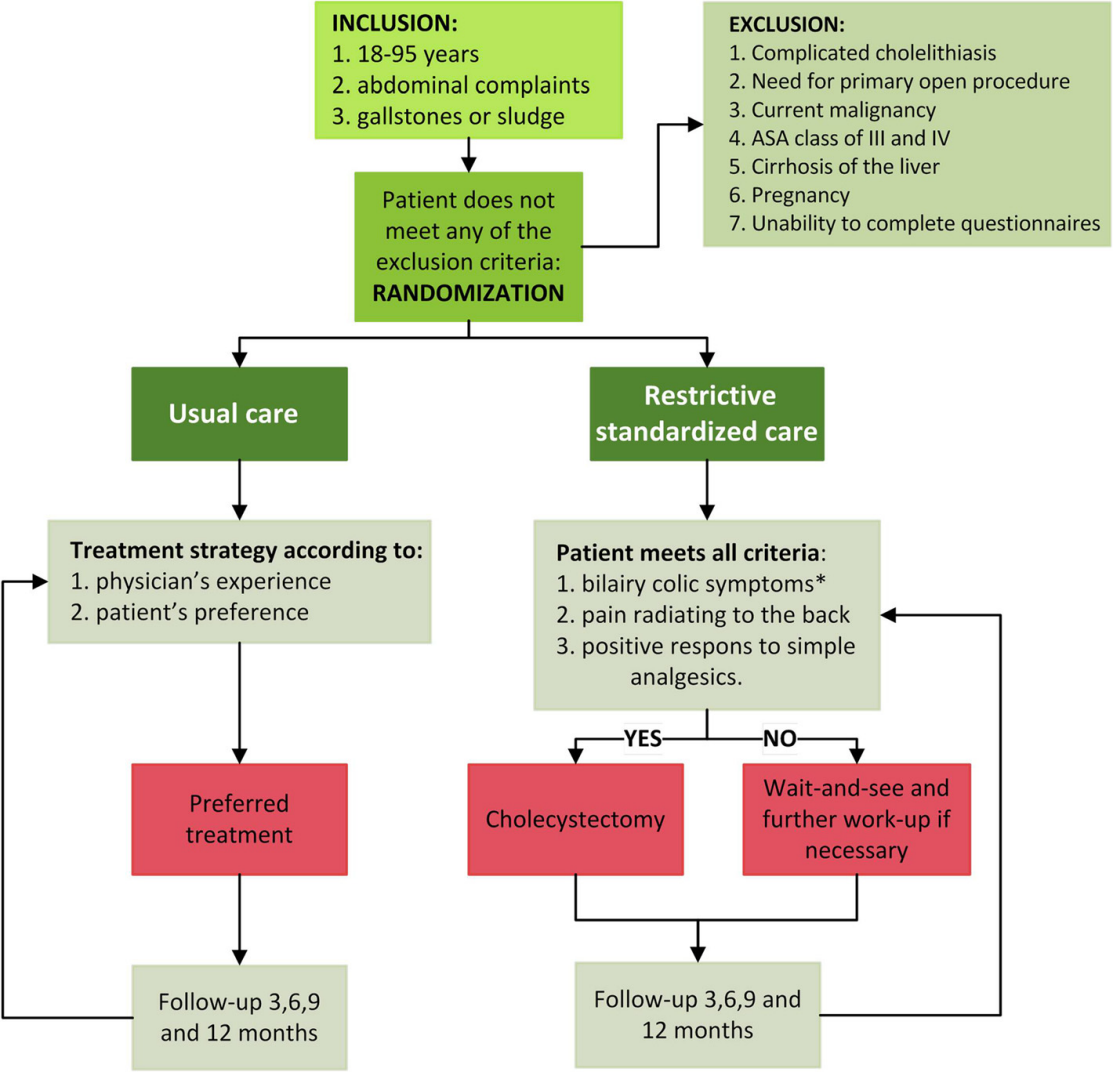
Flow-chart of the inclusion, randomization and management in the SECURE-trial. *Bilairy colic symtoms are defined as severe steady pain, lasting 15–30 min or longer, usually located in the epigastrium and/or right upper quadrant

Patients will be randomized to either usual care or to the restrictive strategy. Inclusion, randomization and management are illustrated in Fig. 1.

### Usual care

Patients assigned to the control group will receive the usual care given at participating centres. During the first visit at the surgical out-patient clinic subjects are seen by a random surgeon, who will assess history, examine the patient and review investigations. Diagnostic and treatment decisions will be based on the physician’s knowledge, preference and experience and on the patients’ preferred choice of treatment. The results of treatment will be evaluated during one year of follow-up using web-based questionnaires.

### Restrictive standardized strategy

The restrictive strategy includes a standardized work-up with stepwise selection for surgery. In this restrictive arm patients are selected for surgery after a specified history using a triage instrument based on the Rome criteria for biliary colic [14, 15] and systematic review of the literature [24, 25]. According to the Rome criteria a biliary colic is defined as a severe steady pain, lasting 15–30 min or longer, usually located in the epigastrium and/or right upper quadrant [14, 15]. The biliary colic defined according to the Rome criteria has shown to be insufficiently accurate for the diagnosis of symptomatic cholecystolithiasis [26]. Systematic reviews of the literature have shown that three symptoms have a significant relationship with the diagnosis of symptomatic cholecystolithiasis: biliary colic, pain radiating to the back and a positive response to simple analgesics [24, 25].

Patients who meet these three criteria: biliary colic (Rome definition), pain radiating to the back and pain reduction after analgesics are selected for primary gallbladder removal. Patients who do not meet all these criteria go for further work-up of alternative diagnoses and have an interval evaluation at the outpatient clinic every three months. This work-up, symptoms and the effect of therapy aimed at another likely diagnosis are repeatedly evaluated every three months during one year of follow-up using web-based questionnaires. At each of the evaluation moments the indication for surgery can be made or conservative approach is continued

The use of diagnostics (e.g., gastroscopy) or therapeutics (e.g., analgesics or antacids) aimed to diagnose or treat other possible diseases causing the abdominal symptoms are left to the discretion of the treating physician.

### Randomization

Patients will be randomized 1:1 between the usual care group and the restrictive care group. Randomization will be computer- and web-based using stratification to ensure a balanced distribution of (un-)known possible confounders in both treatment groups, and in blocks of variable size.

Randomization will be stratified according to the following characteristics: center, sex and body mass index. To ensure allocation concealment the randomization list will be generated using an online computer software program (ALEA NKI-AVL, Amsterdam, The Netherlands, Release: 2.2.) and implemented into a web-based application.

### Sample Size

For the power analysis we assumed that the percentage of pain-free patients in the restrictive arm will be at least equal to the usual care arm at the end of follow-up. For the calculation we assumed, based on literature, that after usual care a maximum of 80 % of patients will be pain-free [13]. If the restrictive strategy results in less than 75 % of patients pain-free, then this strategy will not be considered non-inferior. However, it is hypothesized that the percentage of pain-free patients in the restrictive strategy group will be slightly higher than the percentage in the usual care arm and rise to 82 % or above.

If the usual care arm becomes, to some extent, contaminated by the restrictive strategy arm, then the 80 % pain-free estimate for the usual care arm will tend towards the 82 % for the restrictive standardized work-up arm. If so, the boundary of non-inferiority should be increased as well in order to maintain the non-inferiority of 5 %. Hence, if contamination would result in 81 % of patients pain-free in the usual care arm, then the lower boundary of non-inferiority equals 76 %. Although we do not expect contamination to happen, it is accounted for in this calculation of the sample size. Thus, with a one-sided Z test, 80 % power and a significance level of 5 % a total of 1038 evaluable patients (519 in each arm) needs to be included, if the boundary of non-inferiority equals 76 %. In the absence of contamination and a lower boundary of non-inferiority of 75 %, this total number of 1038 evaluable patients will result in a power of 89 %.

### Cost-effectiveness

The economic evaluation will be undertaken as a cost-effectiveness analysis (CEA) with the costs per patient pain-free at 12 months as primary outcome measure. Additionally, a cost-utility analysis (CUA) will be performed with the costs per quality adjusted life-year (QALY) as outcome. Both analyses will be performed from a societal perspective and the time horizon is set at 12 months.

### Questionnaires

Patients can complete the questionnaires through the trial website or they can complete a paper copy.

The questionnaire set consists of four parts and will be completed at baseline and at 3, 6, 9 and 12 months of follow-up:

- The EuroQol 5 Dimensions (EQ-5D) combined with a Short-Form Health and Labour Questionnaire (SF-HLQ) [27]: The EQ-5D is a generic questionnaire and covers five domains of health (mobility, self-care, usual activities, pain/discomfort and anxiety/depression). The SF-HLQ contains three modules covering absence from paid employment, production loss without absence from paid employment and impediments to paid of unpaid employment. Additional questions assess health care consumption based on home, primary and secondary care consultations, emergency department and hospital admissions, medication use and out-of-pocket costs.
- The Gastrointestinal Quality of Life Index (GIQLI) [28]: This questionnaire includes both specific questions on gastrointestinal symptoms, for both the upper and lower digestive tracts, as well as generic questions on physical, emotional and social capabilities.^26,27^ The GIQLI includes 5 domains: symptoms, physical dysfunction, emotional dysfunction, social dysfunction and treatment-related stress
- The Izbicki Pain Score (IPS) [29]: This questionnaire is designed for upper abdominal pain and based on four questions regarding frequency of pain, intensity of pain (as indicated by a visual analogue score), use of analgesics and disease-related inability to work.
- The Gallstone Symptom List [30]: This specific pain score was designed and previously used in a large cohort of patients in the United Kingdom. This score was designed to asses symptoms associated with symptomatic cholecystolithiasis.

### Safety monitoring

An independent data and safety monitoring committee (DSMC) will be established. This committee will be guided by a charter defining their role and responsibilities, and methods specific to the committee. The DSMC will assess safety by analysis of the complication ratio due to surgery or due to the gallstones in both study arms after 50 % of the patients are included. The DSMC will perform safety assessments consisting of the analysis of proportion of complications due to cholecystectomy in the total number of complications. The choice of appropriate statistical technique, if any, will be left to the discretion of the DSMC, but may consist of a regression analysis on the proportion of complications due to cholecystectomy, with treatment and the proportion of patients with cholecystectomy as covariates. The DMC will advise the study group to continue, to adapt or to terminate the study.

### Statistical anlysis

Analyses will be carried out according to the intention-to-treat principle as well as the per protocol principle. For continuous data, Student’s *t*-test will be used to calculate differences between groups for normally distributed data or Mann-Whitney *U* test for non-normally distributed data. The *χ*^2^ test will be used to compare dichotomized outcomes between the groups.

The generalized estimating equations (GEE) will be used to examine the impact of (i) centre, sex and weight on the probability of being pain-free at 12 months post-randomisation, and (ii) treatment strategy, centre, sex and weight on the number of cholecystectomies. This procedure extends standard regression analysis, taking into account the correlation between measurements. To assess the relation between specific symptoms or sets of symptoms and being pain-free at 12 months post-randomisation, logistic regression analyses will be performed. Data on quality of life will be assessed by repeated measurement analysis using a linear mixed model. In all analyses, statistical uncertainties are expressed in 95 % two-sided confidence intervals. A *p*-value of <0.05 will indicate statistical significance.

### Ethical consideration and informed consent

This trial will be conducted in accordance with the principles of the Declaration of Helsinki and as stated in the laws governing human research in the Netherlands and Good Clinical Practice. The Medical Ethical Committee (MEC) of the Academic Medical Center Amsterdam and local ethical committees of all participating centres have approved this protocol.

This protocol was endorsed by the board of directors of each participating hospital prior to the inclusion of subjects at the respective hospital.

Informed consent will be obtained from each participating patient in oral and written form prior to randomization.

## Discussion

### Rationale of study design

In the present study we chose a pragmatic approach to investigate the effectiveness of a more restrictive care strategy for patients with cholecystolithiasis. The parallel-arm, non-inferiority study design best fits the focus of research in which optimal patient selection (identifying the right patient for the right treatment) rather than treatment effectiveness (identifying the best treatment given the right patient) is studied; estimates of both, the proportion of patients in whom unnecessary cholecystectomies can be prevented as well as the resulting overall effectiveness at the group level, automatically follow from this design. The current study design of randomizing patients to usual care or the restrictive standardized work-up does not exhibit the weaknesses of alternative designs which were considered. First, a design with randomization of patients to laparoscopic cholecystectomy or no surgery would focus exclusively on the effect of surgery itself and would be in need of a well-defined target population as reflected in selective inclusion and exclusion criteria. Such design would ignore the core issue in this proposal that practice variation and overconsumption of care result from the presence of indeterminate means of patient selection and indication. Second, a cluster randomized design with hospitals (rather than patients) randomized to usual care or to the restrictive standardized work-up would run the risk of selection bias and confounding. Selection bias in a cluster randomized trial might occur, if the local recruitment of patients would be influenced by prior knowledge of the treatment strategy offered by the hospital; confounding following an uneven distribution of hospitals with similar characteristics over both trial arms might easily occur given the number of participating hospitals. In addition, an adequate informed consent procedure for such cluster randomized trial would be difficult to accomplish.

A potential pitfall of the present design is the risk of contamination of usual care by the restrictive standardized work-up approach as it is conceivable that specialists transfer their experience with this work-up to usual care. It should be noted however that the equipoise principle still holds for usual care and the standardized work-up. It is very unwise and perhaps even unethical to adjust usual care as long as the clinical non-inferiority and health economic superiority of the restrictive standardized work-up approach has not been demonstrated yet. The equipoise principle should make surgeons in the participating hospitals indifferent regarding their preference for either approach during the study period. Moreover many different surgeons are involved making a strong and uniform contamination effect unlikely.

To further counter the risk of contamination as well as its potential impact on the study results, we will monitor each step during usual care in the clinical report forms. Additionally, a sample size is calculated conditional on the presence of contamination, which will be reevaluated during interim analysis and adjusted if indicated.

To optimally reflect the current health care practice all patients with abdominal pain and gallstones are included in this study. Pre-selection by excluding patients with non-specific abdominal symptoms and only including patients with specific “biliary colic” would potentially optimize health care practice outcome, but would leave optimization efforts unmeasured and would give no insight in the obvious difficulty of this selection. Selected inclusion would certainly hamper the future implementation of results by leaving the dispute unresolved of which symptoms are typical and which are not. This will risk non-compliance to new guidelines as these would not reflect real life. The ‘typical’ gallstone colic patients are less typical than definition suggests. Even if a colic resulting from gallstones is almost certain, then still, wide differences of opinion exist between surgeons of how many colics are needed and which time interval between colics should have presented to indicate surgery.

### Impact of the secure trial

Both the inefficacy of cholecystectomy in a considerable number of patients as well as a substantial practice variation in gallbladder surgery warrant a more evidence-based approach for this high volume procedure. The first challenge in evaluating patients with upper abdominal symptoms found to have gallstones is to determine whether the stones are the cause of the symptoms or merely an incidental finding.

There is no national or international prospective randomized study of the efficiency gains of a standardized work-up and interval evaluation with stepwise selection for laparoscopic cholecystectomy. The best available patient reported data comes from the long term follow up from a randomized study which showed that a watchful waiting strategy was shown to be a feasible option in 31 % of patients with uncomplicated symptomatic cholelithiasis during 14 years of follow-up [31]. In the absence of existing evidence it is clear that a more adequate discriminative selection tool for cholecystectomy is necessary. The present study aims to improve the outcome of patients with gallstones and abdominal complaints by identifying those patients with an absolute indication for cholecystectomy based on an accurate assessment of symptomology. This will ultimately maximise the number of patients successfully treated for biliary pain and minimise not only patients with persistent post cholecystectomy pain, but also the risks of unnecessary operative intervention. Secondly, this study examines the cost-effectiveness and budgetary impact of a more restrictive strategy for cholecystectomy against usual care for patients with abdominal complaints and gallstones. In the present trial an evaluation will be performed on whether a restrictive strategy with standardized work-up, interval evaluation and stepwise selection for surgery can improve patient outcomes with a reduced number of cholecystectomies. Results of the present study as proposed here will affect health care policy concerning cholecystectomy for the coming decades.

### Conclusion

The SECURE-trial is a multicentre trial designed to assess the effectiveness of a more restrictive treatment strategy in patients with gallstones and abdominal complaints, using a restrictive standardized work-up with stepwise selection for cholecystectomy. This trial aims to improve the outcome of patients after cholecystectomies by optimizing the indications for cholecystectomy. Therefore, the SECURE-trial has the potential to impact daily clinical practice and the development of national and potentially international guidelines concerning the indications for cholecystectomy in symptomatic gallstone disease.

### Abbreviations

ASA, a five-category physical status classification system developed by the American Society of Anesthesiologists; CEA, cost-effectiveness analysis; CUA, cost-utility analysis; DSMC, data monitoring committee; GEE, generalized estimating equations; MEC, medical ethical committee; QALY, quality adjusted life-year; SECURE trial, scrutinizing the (in)efficient use of cholecystectomy: a randomized trial; VAS, visual analogue scale
